# Current and Emerging Therapies for C3 Glomerulopathy and Primary (Idiopathic) Immune Complex Membranoproliferative Glomerulonephritis

**DOI:** 10.1016/j.ekir.2025.10.020

**Published:** 2025-11-05

**Authors:** David Kavanagh, Gema Ariceta, Marina Vivarelli, Franz Schaefer, Fernando Caravaca-Fontán, Véronique Frémeaux-Bacchi, Fadi Fakhouri, Christoph Licht, Matthew C. Pickering

**Affiliations:** 1 Faculty of Medical Sciences, Newcastle University, Newcastle upon Tyne, UK; 2 National Renal Complement Therapeutics Centre, Royal Victoria Infirmary, Newcastle upon Tyne, UK; 3 Department of Pediatric Nephrology, University Hospital Vall d’Hebron, Autonomous University of Barcelona, Barcelona, Spain; 4 Laboratory of Nephrology and Clinical Trial Center, IRCCS Bambino Gesù Children’s Hospital, Rome, Italy; 5 Department of Pediatric Nephrology, Center for Pediatrics and Adolescent Medicine, University Hospital Heidelberg, Heidelberg, Germany; 6 Department of Nephrology, Instituto de Investigación Hospital 12 de Octubre, Madrid, Spain; 7 Department of Immunology Biology, Assistance Publique-Hôpitaux de Paris, Hôpital Européen Georges Pompidou, Paris, France; 8 Department of Nephrology and Hypertension, Lausanne University Hospital, Centre Hospitalier Universitaire Vaudois, Lausanne, Switzerland; 9 Department of Pediatrics, The Hospital for Sick Children and University of Toronto, Toronto, Ontario, Canada; 10 Department of Immunology and Inflammation, Imperial College, London, UK

**Keywords:** C3G, complement 3 glomerulopathy, complement inhibition, complement system, IC-MPGN, immune complex membranoproliferative glomerulonephritis

## Abstract

C3 glomerulopathy (C3G) and primary (idiopathic) immune complex membranoproliferative glomerulonephritis (IC-MPGN) are rare kidney diseases characterized by dysregulation of the complement system and progressive deposition of C3 and its breakdown products in the glomeruli, ultimately leading to kidney failure in up to 50% of patients within 10 years. Until recently, standard approaches to treatment included supportive measures common to many kidney diseases and immunosuppression to mitigate inflammation, rather than specific therapies addressing the underlying C3 dysregulation. However, recent advances in targeted complement inhibitor therapy have been made in these diseases with positive results from phase 3 clinical trials of both the factor B inhibitor, iptacopan (in adults with native kidney C3G) and the C3/C3b inhibitor, pegcetacoplan (in adults and adolescents with native or posttransplant C3G or primary IC-MPGN). In this review, we summarize what is known and what questions still remain regarding the effect of complement inhibitors on widely accepted surrogate end points for efficacy in C3G/primary IC-MPGN (proteinuria, estimated glomerular filtration rate [eGFR], and kidney biopsy histology). Additional controversies, including candidate patient populations, optimal treatment duration, and how best to monitor patients on complement inhibitor therapy are also discussed, in an effort to prepare the nephrology community for innovative therapeutic options for patients whose long-term prognosis has generally been dismal.

C3G and primary or idiopathic IC-MPGN (hereafter referred to as primary IC-MPGN) are rare kidney diseases with the incidence of C3G in the USA and Europe variably estimated as 0.2 to 3 cases per million and prevalence of 0.05 to 1.4 cases per 10,000.^1^

Dysregulation of the complement system is the primary driver of disease in C3G and primary IC-MPGN. Specifically, overactivation of the complement cascade leads to the deposition and accumulation of C3 breakdown products along the glomerular basement membrane and within the mesangium, triggering glomerular injury and contributing to progressive kidney disease. C3G and primary IC-MPGN can appear identical on light microscopy; however, C3G is diagnosed when immunofluorescence staining for C3 deposits is dominant relative to other immune reactants, whereas in primary IC-MPGN, there is codominance of C3 and immune complex deposition (IgG, C1q, IgA, and/or IgM). True primary IC-MPGN is rare, particularly in adults, making its pathogenesis more unclear; however, it is associated with serologic or genetic evidence of complement dysregulation at a similar frequency to C3G, leading to the C3 overactivation common to both diseases.^1^, ^2^, ^3^, ^4^

Potential triggers of complement dysregulation include autoantibodies targeting complement proteins and, rarely, pathogenic variants in genes encoding for components or regulators; in many cases, the cause is unknown. Mouse models have shown that deficiency of factor H (a regulator of C3 convertase formation and decay) leads to spontaneous development of C3G; this phenotype in mice may be partially rescued by inactivation of C5 and the terminal complement pathway, or completely rescued by C3 or factor B depletion.^5^^,^^6^ Familial C3G is very rare, but includes complement factor H-related protein 5 nephropathy,^7^ factor H deficiency,^8^ gain-of-function mutations in either *CFB*^9^ or *C3*,^10^ and complex gene rearrangements within the *FHR* gene cluster.^1^ Rare variants in complement-related genes are present in ≤ 20% of patients and some series report associations with poor outcomes.^11^ However, in a large study of C3G and primary IC-MPGN, it was human leukocyte antigen type, not rare complement gene variation, that was associated with disease suggesting an underlying autoimmune mechanism in most cases.^12^

Autoantibodies targeting neoepitopes within complement convertases (nephritic factors [NeFs]) are present in 40% to 80% of C3G and primary IC-MPGN patients,^2^^,^^13^ which cause convertase stabilization and increase activation of key complement proteins C3 and C5.^2^^,^^14^ How NeFs are relevant to the pathogenesis of kidney disease has long been disputed.^15^^,^^16^ For example, NeFs are frequently seen with hypocomplementemia in partial lipodystrophy in the absence of any kidney disease,^17^^,^^18^ have been reported in isolated healthy individuals^19^^,^^20^ and can be transiently seen during postinfectious glomerulonephritis.^21^ It is not known whether NeFs arise as a consequence of uncontrolled C3 convertase formation or are primary drivers of C3 dysregulation.

C3 deposits in both diseases ultimately lead to progressive inflammation, disrupting glomerular function.^1^^,^^2^ Glomerular damage manifests as proteinuria and hematuria in most patients. The heterogeneous nature of these diseases is reflected in the clinical presentation, where severity of manifestations at onset can range from asymptomatic microscopic hematuria to nephrotic syndrome or advanced chronic kidney disease.^1^^,^^22^^,^^23^

Although C3G outcomes in pediatrics can be favorable,^24^ the generally poor prognosis for C3G and primary IC-MPGN has persisted because available treatments do not target the underlying C3 dysregulation. Treatments have focused on nonspecific approaches of key importance in many kidney diseases such as optimization of blood pressure and proteinuria control.^25^^,^^26^ Where there are inflammatory changes within biopsies, immunosuppression has been used, most commonly glucocorticoid therapy and mycophenolate mofetil, with escalation to B-cell depletion with rituximab considered in IC-MPGN patients with no improvement.^23^^,^^25^^,^^27^ However, many immunosuppressive treatments in kidney disease have a narrow therapeutic window, patients must be closely monitored because of a high risk of adverse events,^28^ and their effectiveness is variable and often associated with relapse once patients taper off treatment (based on a retrospective study).^29^

Randomized controlled trials of novel therapies aimed at preventing C3 activation have been conducted in C3G and primary IC-MPGN (both together and separately, depending on the trial), with highly encouraging reports of early efficacy and safety from complement C3/C3b– (in both diseases) and factor B–directed inhibitors (in C3G).^30^, ^31^, ^32^, ^33^, ^34^, ^35^, ^36^ In addition, there are many ongoing or planned trials for other therapies, including ribonucleic acid interference of C3, dual inhibition of factor H and C5, and inhibition of mannan-binding lectin-associated serine proteases 2 and 3.^2^^,^^37^ In this review, we discuss the known and controversial aspects of targeted complement inhibition in C3G and primary IC-MPGN, and frame future clinical and research questions for the nephrology community.

## Evaluating The Efficacy of Targeted Therapies

There has been a need in C3G and primary IC-MPGN to determine the regulatory approval pathway for novel targeted therapies. Because kidney failure typically occurs years after diagnosis, clinical trials investigating efficacy in this population necessarily use surrogate end points of kidney failure. Until recently, there were no validated surrogate end points for these conditions, so the focus had been on defining candidate and reasonably likely surrogate end points through analyzing retrospective cohort data and natural history studies. This is a challenging task in diseases that are rare and have quite marked heterogeneity in clinical outcome. Nevertheless, significant progress has been achieved through the study of multiple registries. The Kidney Health Initiative convened an expert working group to provide guidance on clinical trial efficacy end points in C3G. The working group agreed that evidence from natural history studies supports proteinuria, eGFR, and histopathology as relevant biomarkers for measuring disease progression or activity.^38^

Longitudinal change in proteinuria appears to be a good marker for risk of disease progression in C3G and primary IC-MPGN.^38^, ^39^, ^40^, ^41^, ^42^

eGFR, particularly the rate of change in eGFR over time (the “eGFR slope”), is regarded as an informative measure of kidney function when measured long term.^38^^,^^40^, ^41^, ^42^

From our understanding of the pathophysiology, abnormal C3 activation is the key step in triggering kidney injury and diagnosis of C3G and primary IC-MPGN relies upon immunofluorescence staining for C3.^2^^,^^25^ Targeted agents that stop C3 activation would be predicted to reduce or resolve C3 deposition in the kidney and changes in biopsy C3 staining over time have been analyzed in recent phase 3 studies to investigate whether these agents are preventing C3 breakdown and accumulation within glomeruli.^31^^,^^38^^,^^43^ With effective therapy, histologic index scoring systems for disease activity and chronicity based on morphological changes should identify temporal changes in glomerular inflammation (e.g., reduction or resolution of glomerular macrophages) and reveal the extent of reversibility of chronic lesions in the mesangial matrix and glomerular basement membrane.^22^^,^^44^ However, recent clinical trial data suggest that they may be less effective than initially expected for assessing treatment response at 26 weeks,^30^^,^^31^ and the reliability and responsiveness to change of the histologic index remain unclear.^38^

The Kidney Health Initiative working group agreed that demonstrating a favorable effect on proteinuria, eGFR, and kidney biopsy histology would provide convincing evidence of treatment efficacy in C3G.^38^ Nevertheless, it will be essential to confirm that favorable changes in these parameters in the timeframe typically reported in trials (e.g., 1 year) associate with prevention (or significant reduction) in kidney failure long-term.

## Inhibitors of Terminal Complement and Related Components in C3G/Primary IC-MPGN: Eculizumab and Avacopan

**Figure 1:**
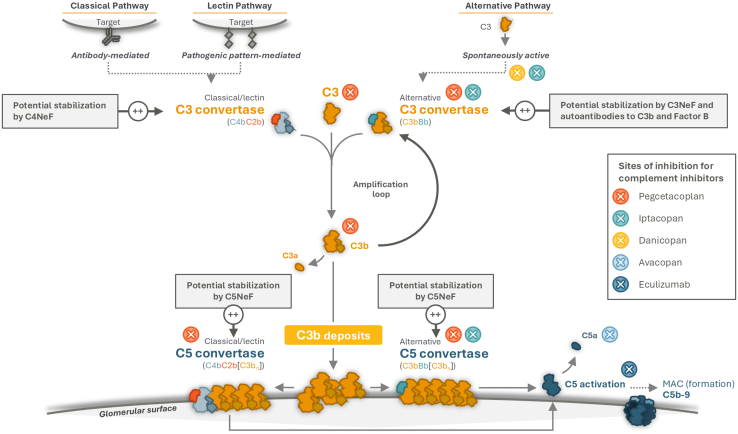
The complement cascade, the influence of NeFs or autoantibodies to complement components, and targets for key complement inhibitors. MAC, membrane attack complex; NeF, nephritic factor.

Eculizumab is a monoclonal anti-C5 antibody that prevents cleavage of C5 into its active components C5a and C5b; it therefore stops membrane attack complex formation (Figure 1). Eculizumab has been used in patients who have failed to respond to immunosuppression and glucocorticoids, and despite reports of responses to eculizumab particularly in crescentic rapidly progressive C3G,^45^, ^46^, ^47^, ^48^, ^49^ the overall body of data supporting its use in either IC-MPGN or C3G is unconvincing.^24^^,^^46^^,^^48^, ^49^, ^50^, ^51^, ^52^, ^53^

Avacopan is a small molecule antagonist of the C5a receptor, which blocks C5a-mediated neutrophil activation. The phase 2 randomized, placebo-controlled ACCOLADE trial did not meet its primary end point of change in C3G Histologic Index for disease activity and showed no significant difference between avacopan and placebo in key secondary end points of proteinuria and eGFR after 52 weeks of treatment.^54^

Taken together, the experience of inhibition at the level of C5 in these conditions has taught us that though it can reduce terminal complement pathway–mediated glomerular inflammation, the unhindered activation of C3 remains untreated. Inhibitors targeting complement components involved in C3 activation are thought to be a more rational approach to therapy.

## Proximal Complement Inhibitors in C3G/Primary IC-MPGN: Danicopan, Iptacopan, and Pegcetacoplan

Danicopan is a small molecule inhibitor of factor D, a protease that mediates an essential step in formation of the alternative pathway C3 convertase that activates C3. It was studied in 2 phase 2 trials that failed to meet their efficacy end points, likely because of incomplete and inadequate sustained inhibition of the alternative C3 convertase.^55^

Iptacopan is a small molecule inhibitor of factor B, which blocks formation of the alternative pathway C3 convertase^35^ and has been approved to treat complement-related diseases, paroxysmal nocturnal hemoglobinuria^56^ and, in the USA, IgA nephropathy.^57^ In 2025, iptacopan was also approved by the US Food and Drug Administration for the treatment of adults with C3G to reduce proteinuria,^58^ and by the European Medicines Agency for the treatment of adults with C3G in combination with a renin-angiotensin-aldosterone system inhibitor or as monotherapy in patients who are renin-angiotensin-aldosterone system inhibitor-intolerant.^59^ Iptacopan is administered orally at a recommended dose of 200 mg twice daily.^58^^,^^59^

A phase 2, single-arm trial of oral iptacopan in native kidney or posttransplant C3G patients (NCT03832114) demonstrated reductions in proteinuria after 12 weeks in the native cohort, with improvement or stabilization of eGFR and reduction in median C3 deposit score of 2.5 (on a scale of 0–12) in the transplanted cohort after 12 weeks of treatment.^35^ Iptacopan increased serum C3 and reduced sC5b-9 levels, although not all patients had normalization of C3 levels.^35^^,^^36^

In the phase 3 APPEAR-C3G trial (NCT04817618), 74 adult patients with native kidney C3G were randomized to receive iptacopan or placebo for 26 weeks, followed by an open-label period of continued iptacopan treatment or a switch from placebo to iptacopan for another 26 weeks. The primary end point was change from baseline in proteinuria (measured by 24-hour urine protein-to-creatinine ratio [UPCR]) at 26 weeks.^30^^,^^60^ A second cohort of adolescent patients with C3G (aged ≥ 12 years) in APPEAR-C3G and a dedicated phase 3 trial for patients with primary IC-MPGN (APPARENT; NCT05755386) are ongoing based on positive phase 3 results in the adult C3G population.^61^

Pegcetacoplan is a PEGylated cyclic peptide that selectively binds to C3 and C3b, blocking C3 activation from both alternative and classical/lectin pathway C3 convertases, as well as formation of the alternative pathway C3 convertase.^62^ Pegcetacoplan has been approved to treat paroxysmal nocturnal hemoglobinuria^63^ and, in the USA, geographic atrophy secondary to age-related macular degeneration.^64^ In 2025, pegcetacoplan was also approved by the US Food and Drug Administration for the treatment of adult and pediatric patients aged ≥ 12 years with C3G or primary IC-MPGN to reduce proteinuria.^65^ Pegcetacoplan is administered as a subcutaneous injection at a recommended dose of 1080 mg (in 20 ml) twice a week for adults using a commercially available infusion pump or on-body injector device, where available. Weight-based dosing is recommended for pediatric patients.^65^

Pegcetacoplan has been investigated in phase 2 trials for both C3G and primary IC-MPGN, where it was hypothesized that blocking both classical/lectin and alternative pathways at the C3/C3b level would be similarly effective for both diseases. Results from phase 2 trials showed reductions in proteinuria and eGFR improvement or stabilization over up to 52 weeks of treatment.^32^, ^33^, ^34^ In the phase 2 randomized controlled NOBLE trial of pegcetacoplan in posttransplant recurrent C3G or primary IC-MPGN patients (NCT04572854), all pegcetacoplan-treated patients achieved normal-or-higher serum C3 and reduced sC5b-9 levels,^32^^,^^33^ whereas 55% (6/11) and 33% (3/9) of patients demonstrated no glomerular C3 staining and absent electron microscopy deposits, respectively, at 52 weeks.^33^

In the phase 3 VALIANT trial (NCT05067127), 124 adult or adolescent patients (aged ≥ 12 years) with C3G or primary IC-MPGN in native kidney or posttransplant recurrent disease were randomized to receive pegcetacoplan or placebo for a period of 26 weeks, followed by an open-label period of continued pegcetacoplan treatment or a switch from placebo to pegcetacoplan for another 26 weeks. The primary end point was change from baseline in proteinuria (measured by first-morning urine UPCR) at 26 weeks.^31^

Both APPEAR-C3G and VALIANT required patients to have baseline UPCR ≥ 1 g/g (or proteinuria ≥ 1 g/d for VALIANT) and eGFR ≥ 30 ml/min per 1.73 m^2^, with supportive care treatments permitted, provided patients were on a stable dose.^31^^,^^60^ Although low serum C3 levels were mandatory in APPEAR-C3G, this was not a criterion in VALIANT, but active disease parameters were requested. Baseline characteristics for enrolled patients are shown in Table 1.^66^

**Table 1:** VALIANT and APPEAR-C3G trials — baseline characteristics and topline results at week 26

Baseline characteristics	VALIANT^31^		APPEAR-C3G^30^^,^^66^	
	Pegcetacoplan (*N* = 63)	Placebo (*N* = 61)	Iptacopan (*N* = 38)	Placebo (*N* = 36)
Age, yrs, mean (SD)	28.2 (17.1)	23.6 (14.3)	26.1 (10.4)	29.8 (10.8)
Adolescents (12–17 yrs old), *n* (%)	28 (44.4)	27 (44.3)	NA	NA
Sex, female, *n* (%)	37 (58.7)	33 (54.1)	11 (28.9)	16 (44.4)
Race, white, *n* (%)	45 (71.4)	46 (75.4)	27 (71.1)	24 (66.7)
Baseline 24-h UPCR, g/g, mean (SD)	3.95 (2.89)	3.29 (2.36)	3.85 (2.29)	2.93 (1.71)
Baseline eGFR, ml/min per 1.73 m^2^, mean (SD)	78.5 (34.1)	87.2 (37.2)	89.3 (35.2)	99.2 (26.9)
Underlying disease based on screening biopsy, *n* (%)				
C3G	51 (81.0)	45 (73.8)	38 (100)	36 (100)
C3GN	45 (71.4)	41 (67.2)	26 (68.4)	32 (88.9)
DDD	4 (6.3)	4 (6.6)	9 (23.7)	1 (2.8)
Mixed C3GN/DDD	-	-	2 (5.3)	2 (5.6)
Undetermined	2 (3.2)	0	1 (2.6)	1 (2.8)
Primary IC-MPGN	12 (19.0)	16 (26.2)	NA	NA
Posttransplant recurrent disease, n (%)	5 (7.9)	4 (6.6)	NA	NA

Topline results at week 26	VALIANT^31^^,^^65^		APPEAR-C3G^30^^,^^58^^,^^66^	
	Pegcetacoplan (*N* = 63)	Placebo (*N* = 61)	Iptacopan (*N* = 38)	Placebo (*N* = 36)
Relative reduction in proteinuria at week 26^a^, % (95% CI)	−68.1 (57.3, 76.2), p<0.0001		−35.1 (13.8, 51.1), *P* = 0.0014	
Patients who achieved ≥ 50% reduction in proteinuria at week 26, *n* (%)	38 (60.3)	3 (4.9)	11 (29.7)	2 (5.6)
Patients who achieved < 1 g/g UPCR at week 26, *n* (%)	32 (50.8)	12 (19.7)	4 (10.5)	2 (5.6)
Mean difference in eGFR at week 26, ml/min per 1.73 m^2^ (95% CI)	+6.3 (0.5, 12.1), *P* = 0.03^b^		+2.2 (-2.8, 7.1), *P* = 0.324	
Evaluable patients with reduced C3 renal biopsy staining at week 26, n/N (%)	26/35 (74.3)	4/34 (11.8)	NR	
Patients who achieved composite renal end point at week 26, *n* (%)^c^	31 (49.2)	2 (3.3)	11 (29.7)	2 (5.6)
Summary of safety outcomes at week 26	• Majority of TEAEs mild-to-moderate in severity (95.2% in the pegcetacoplan arm) • Most common adverse events (≥10% of patients) in the pegcetacoplan arm: o Infusion site reactions (25%) o Pyrexia (19%) o Nasopharyngitis (18%) o Influenza (11%) o Nausea (10%) o Cough (10%) • Serious infections occurred in 4.8% of patients in the pegcetacoplan arm^d^ • No cases of encapsulated meningococcal infection • No deaths related to study treatment		• Majority of TEAEs mild-to-moderate in severity (94.7% in iptacopan arm) • Most common adverse events (≥10% of patients) in the iptacopan arm: o Viral infections (29%) o Nasopharyngitis (11%) • Serious infections occurred in 5.2% of patients in the iptacopan arm^e^ • No cases of meningitis and/or meningococcal sepsis • No deaths related to study treatment	

C3G, complement 3 glomerulopathy; CI, confidence interval; eGFR, estimated glomerular filtration rate; FMU, first-morning spot urine; C3GN, C3 glomerulonephritis; DDD, dense deposit disease; IC-MPGN, immune complex membranoproliferative glomerulonephritis; NA, not applicable; NR, not reported; TEAE, treatment-emergent adverse event; UPCR, urine protein-to-creatinine ratio.

a Between pegcetacoplan and placebo in VALIANT or iptacopan and placebo in APPEAR-C3G. FMU UPCR for VALIANT and 24h-UPCR for APPEAR-C3G.

b Nominal *P*-value. Statistical testing stopped after first endpoint to not reach significance between treatment arms.

c ≥ 50% reduction in UPCR + ≤ 15% reduction in eGFR.

d One patient with COVID-19 pneumonia, one patient with influenza, and one patient with pneumonia.

e One patient with pneumonia and bacteremia secondary to an encapsulated organism (*S. pneumoniae*), one patient with an infected bite.

## Evidence of Efficacy from the APPEAR-C3G and VALIANT Phase 3 Trials

### Proteinuria

Change from baseline to week 26 in proteinuria, as measured by UPCR, was the primary efficacy end point for both APPEAR-C3G and VALIANT. Both studies met their primary end points, demonstrating significant improvement in proteinuria compared to placebo (Table 1). Across adult patients with C3G, iptacopan led to a 35.1% mean reduction in proteinuria from baseline relative to placebo (*P* = 0.0014), where mean reduction from baseline in the treated arm was 30.2%.^30^ Across adult and adolescent patients with C3G and primary IC-MPGN with native disease or posttransplant disease recurrence, pegcetacoplan led to a 68.1% mean reduction from baseline relative to placebo (*P* < 0.0001), where mean reduction from baseline in the treated arm was 67.2%.^31^

In addition, in APPEAR-C3G 29.7% (11/37) of iptacopan-treated patients achieved a ≥ 50% reduction in proteinuria compared with 5.6% (2/36) for placebo,^30^ whereas VALIANT reported that 60.3% (38/63) of pegcetacoplan-treated patients achieved ≥ 50% reduction compared with only 4.9% (3/61) receiving placebo.^31^

After 26 weeks of therapy, 10.5% of patients (4/38) had achieved UPCR < 1 g/g with iptacopan^30^ compared with 50.8% (32/63) of patients achieving UPCR < 1 g/g with pegcetacoplan.^31^

In the VALIANT trial, analyses of proteinuria reduction at week 26 for prespecified subgroups of adults (relative reduction: 62.5%), adolescents (74.5%), native (67.5%), and transplanted kidney (64.9%), C3G (65.8%), primary IC-MPGN (73.7%), and patients with (70.3%) or without immunosuppression (64.5%) were consistent with the full cohort. Only adult C3G native kidney patients were enrolled in the APPEAR-C3G trial^31^^,^^67^; however, proteinuria reduction at week 26 appeared to be greater in patients with C3G who were not receiving immunosuppression at baseline (relative reduction: 47.5%) compared with those who were (14.5%).^66^

Open-label extension analyses of APPEAR-C3G and VALIANT have shown that the mean levels of proteinuria reduction achieved after 26 weeks of iptacopan or pegcetacoplan therapy are maintained in patients who complete 52 weeks of therapy.^68^^,^^69^

#### Outstanding Questions

Although it is apparent that both iptacopan and pegcetacoplan reduced proteinuria, it remains unclear what magnitude of proteinuria reduction is clinically meaningful. For both C3G and primary IC-MPGN, a ≥ 50% proteinuria reduction after 12 months has consistently been associated with significantly lower risk of kidney failure, as demonstrated by both the UK RaDaR and Spanish GLOSEN registries. More modest benefits are predicted for patients with lower (≥ 30%) proteinuria reduction at 6 months or 12 months.^41^^,^^70^

In terms of a therapeutic target value for proteinuria, it has been shown that patients who achieve a UPCR < 0.88 g/g (< 100 mg/mmol) at 12 months after diagnosis benefit from a 90% lower risk of kidney failure than those who did not achieve this threshold. In this analysis of 371 patients with C3G and primary IC-MPGN with retrospective and prospective data collection, it was demonstrated that the greatest reductions in risk were achieved below the 0.88 g/g threshold, with an attenuation of the relative benefits as 12-month proteinuria increased.^41^ Altogether, these data point to a spectrum of benefit to proteinuria reduction in which greater reductions are associated with greater likelihood of long-term benefit.

Proteinuria treatment targets may be pertinent to complement inhibitor therapy choice considering the large difference in relative proteinuria reduction observed between APPEAR-C3G and VALIANT. However, it is important to remember that comparisons between trials should be approached with caution because of differences in study designs and patient populations.

Ultimately, treatment targets are likely to be patient-specific, where the degree of chronic kidney damage and the baseline level of proteinuria will dictate what improvement may reasonably be expected from complement inhibitor therapy. To this end, methods for estimating residual proteinuria arising from irreversible glomerular and/or tubulointerstitial scarring will be beneficial in setting tailored goals.

No data is available on patients that were excluded from the trials, in particular patients with > 50% glomerular sclerosis and eGFR ≤ 30 ml/min per 1.73 m^2^. Future efforts should focus on the collection of data from this patient subgroup to allow a better evaluation of treatment response in real-world clinical practice.

### eGFR

A key secondary outcome for APPEAR-C3G and VALIANT was change from baseline to week 26 in eGFR, assessing the impact of therapy on kidney function. In APPEAR-C3G, patients receiving iptacopan displayed stable eGFR after 26 weeks of iptacopan (mean change from baseline +1.3 ml/min per 1.73 m^2^), which was not significantly different from the placebo arm, where eGFR remained stable (mean change from baseline −0.9 ml/min per 1.73 m^2^ for an adjusted mean difference of +2.2 ml/min per 1.73 m^2^; *P* = 0.324).^30^ In VALIANT, patients in the pegcetacoplan arm had stable eGFR (mean change from baseline of −1.5 ml/min per 1.73 m^2^), which was notably different from placebo (mean change from baseline of −7.8 ml/min per 1.73 m^2^ for an adjusted mean difference of +6.3 ml/min per 1.73 m^2^; nominal *P* = 0.03), indicating a protective effect in favor of pegcetacoplan.^31^

As an exploratory end point, APPEAR-C3G additionally reported change in kidney function for each treatment arm by comparing the historical eGFR slope (based on up to 2 years of pretreatment serum creatinine values) to the eGFR slope observed during the trial. Prior to iptacopan, the patients were estimated to have an annual eGFR decline of −10.8 ml/min per 1.73 m^2^. Following iptacopan treatment, estimated annual eGFR change improved significantly to −0.03 ml/min per 1.73 m^2^. eGFR slope also numerically improved in the APPEAR-C3G placebo arm, although to a lesser degree than in the iptacopan arm (from historical annual eGFR change of −7.6 ml/min per 1.73 m^2^ to postplacebo annual eGFR change of −3.1 ml/min per 1.73 m^2^).^66^

#### Outstanding Questions

How complement inhibitor therapy translates to preservation of kidney function is the main clinical outcome of interest in the long-term. The predictive power of eGFR slope early in the disease course is more modest than for change in proteinuria,^41^ indicating a need to assess eGFR slope over longer follow-up periods to be able to detect meaningful clinical changes in kidney function in response to therapy^38^ (e.g., > 12 months).

In this context, even stabilization of eGFR may be considered a success after 6 months of complement inhibitor therapy, although it must be acknowledged that changes in eGFR are dependent on factors such as disease chronicity. Indeed, evidence for eGFR varying by patient population can be seen in the wide range of performance between placebo groups of different clinical trials; change from baseline in eGFR after 26 weeks of placebo in APPEAR-C3G was −0.9 ml/min per 1.73 m^2^,^30^ −3.0 ml/min per 1.73 m^2^ in the ACCOLADE study of avacopan,^54^ −6.9 ml/min per 1.73 m^2^ in the phase 2 study of danicopan,^55^ and −7.8 ml/min per 1.73 m^2^ in VALIANT.^31^ Thus, stabilization of eGFR in randomized controlled trials should be within the context of comparison with the untreated group for the most appropriate interpretation of whether change in eGFR slope is the result of complement inhibitor therapy or of natural disease progression.

Another important unknown quantity is the long-term recovery potential of the kidney. Similar to proteinuria reduction, the potential for eGFR improvement will be patient-specific, factoring in historical kidney function, time since diagnosis, individual nephron endowment. and the degree of irreversible renal parenchymal damage compared with acute severe inflammation.

### C3 Staining

In the VALIANT trial, the effect of treatment on C3 deposition was assessed by immunofluorescence staining for C3c using a polyclonal antibody able to detect C3, C3b, iC3b and C3c (unpublished data), C3 breakdown products and markers of ongoing C3 activation, on kidney biopsies in adult patients. C3 staining was scored on a scale of 0 to 3 (including 0, 1+, 2+, 3+) that has been previously tested in a large, well-defined C3G cohort and recommended for use in clinical practice by an expert working group.^71^^,^^72^ Pegcetacoplan treatment resulted in 74.3% (26/35) of patients achieving a meaningful reduction in staining (≥ 2 point improvement on the 0–3 scale) by week 26 and 71.4% (25/35) had zero C3 staining in the biopsy after treatment.^31^

APPEAR-C3G used a polyvalent antibody for immunofluorescence, also staining for C3b, iC3b, and C3c breakdown products. A modified 12-point glomerular C3 deposition scoring system was used, where staining intensity was initially graded 0 to 3 in the mesangium and capillary separately, with each region granted a deposition extent multiplication factor of 1 (for segmental) or 2 (for global extent). Multiplied scores for each region were summed for a final scoring range of 0 to 12. Iptacopan reduced the mean C3 deposit score by −0.8 from a baseline of 9.2, whereas the score increased by +1.1 from a baseline of 9.6 with placebo after 26 weeks. Mean C3 deposit score improved in both capillary and mesangial regions with iptacopan therapy.^43^^,^^58^

#### Outstanding Questions

Little is known about the evolution of C3 staining in these diseases, because it is not routine practice to repeat biopsies in native disease. Few cohorts have longitudinal data and kidney biopsy analyses to date have typically assessed the prognostic value at diagnosis of gross histopathological features, but not of C3 staining.^22^^,^^44^^,^^73^ However, limited reports of patients with serial biopsies suggest that C3 staining patterns may vary over time, with individual patients shifting from C3G to primary IC-MPGN pathologic features and *vice versa*.^71^^,^^74^ A more recent study of posttransplant C3G patients found that immunofluorescence staining (combined with electron microscopy) was a sensitive means of identifying early disease recurrence, even before overt changes in histology and proteinuria were detectable.^75^

It seems intuitive to assume that because abnormal C3 activation is central to the pathogenesis of these conditions, the ability of a therapy to stop glomerular C3 activation and clear C3 deposits is likely to be a prerequisite for protecting the kidney over the long-term. However, we do not know what degree of C3 inhibition will be needed. In conditions such as paroxysmal nocturnal hemoglobinuria, complement activity must be completely inhibited to stop red blood cell lysis.^76^ This may be the case for C3G and primary IC-MPGN too, but it might also transpire that partial reductions in glomerular C3 have a beneficial impact on kidney function. These questions can only be answered by longitudinal assessment of patients on iptacopan and pegcetacoplan.

## Candidate Patients for Complement Inhibitor Therapy

Based on the phase 3 trial data, the availability of iptacopan and pegcetacoplan may transform the outcome for patients with C3G and primary IC-MPGN, enabling physicians to reduce or stop using glucocorticoids and nontargeted immunosuppression.

However, a prespecified analysis of APPEAR-C3G suggests that patients treated with nontargeted immunosuppressants experience smaller proteinuria reductions with iptacopan. In patients receiving stable doses of immunosuppressive therapy (*n* = 33/74), iptacopan was associated with a 14.5% reduction from baseline in proteinuria relative to placebo compared with a 47.5% reduction in patients not treated with immunosuppressants.^66^ This difference may be linked to a more treatment-resistant nature of C3G in this subgroup.^59^

In the VALIANT trial of pegcetacoplan, proteinuria reduction was similarly robust across all prespecified subgroups (adults [*n* = 69] vs. adolescents [*n* = 55]; native [*n* = 115] vs. transplanted kidney [*n* = 9], C3G [*n* = 96] vs. primary IC-MPGN [*n* = 28]), ranging from 62.5% to 74.5% relative reductions compared to placebo.^31^ Importantly, proteinuria reduction was also consistent with pegcetacoplan regardless of whether patients were treated with immunosuppression at baseline or not. Patients treated with concomitant immunosuppressants (*n* = 90/124) had a relative proteinuria reduction of 70.3% with pegcetacoplan compared to placebo (nominal *P* < 0.0001) and a relative eGFR improvement of +6.8 ml/min per 1.73 m^2^ at 26 weeks, suggesting that pegcetacoplan may be effective in a broad patient population.^77^

#### Outstanding Questions

**Figure 2:**
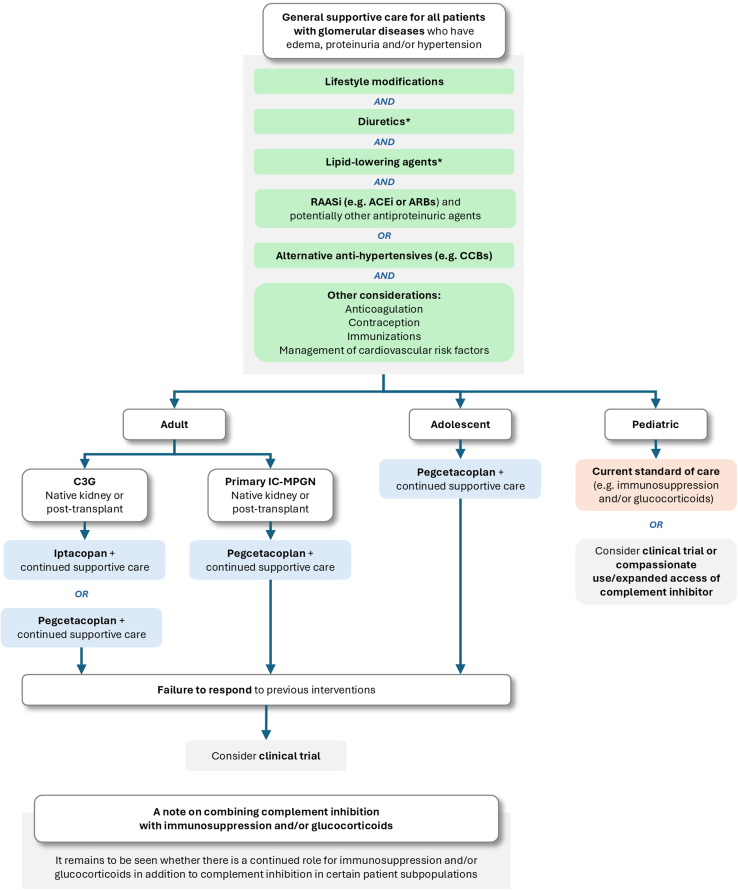
Potential new treatment pathway for the management of C3G and primary IC-MPGN based on available evidence. ∗If required. ACEi, angiotensin-converting enzyme inhibitors; ARB, angiotensin-receptor blockers; C3G, complement 3 glomerulopathy; CCB, calcium channel blocker; IC-MPGN, immune complex membranoproliferative glomerulonephritis; RAASi, renin-angiotensin-aldosterone system inhibitor.

Should all patients with C3G and primary IC-MPGN receive either iptacopan or pegcetacoplan as first-line treatment on top of renin-angiotensin-aldosterone system inhibitor, replacing current immunosuppression? Although supportive care may be sufficient for a subset of patients with mild and stable disease,^2^^,^^24^ the phase 3 trial data suggest that we should be using these agents in patients with significant proteinuria at the outset (Figure 2). The choice of proximal complement inhibitor will evolve as the availability of published data from ongoing trials increases. As in any glomerular disease, it remains crucially important to optimize supportive care. The remaining place of immunosuppressive and antiinflammatory drugs in C3G, particularly forms with significant inflammatory changes, is still to be determined. The duration and necessity of combination therapy with complement inhibitors and immunosuppressants will only become evident as we gather more real-world experience with iptacopan and pegcetacoplan. At this stage we can speculate that in patients with rapidly progressive glomerulonephritis, a group excluded from the trials, immunosuppression (e.g., i.v. glucocorticoid therapy; pulse cyclophosphamide therapy) will continue to be used in the induction phase of treatment, with the rationale of rapidly reducing glomerular inflammation. However, in nonrapidly progressive forms of disease, improvement with complement inhibition was observed in the trial participants who were already on stable immunosuppression at trial entry. This suggests that using complement inhibition as monotherapy at diagnosis and avoiding nonspecific immunosuppression may be the strategy of choice.

Whether initiating complement inhibition in early phases of disease is optimal and, conversely, the therapeutic role of complement inhibition in patients with significant glomerulosclerosis and/or reduced eGFR, remains to be elucidated.

A very important consideration is whether the mechanism underlying C3 dysregulation will impact the selection of inhibitor. Will a patient respond to one drug and not another? Complement inhibitor selection may be influenced by route of administration, where patient preference should be taken into account to ensure there is adequate adherence and to maximize treatment effectiveness.

Finally, it is important to acknowledge that randomized controlled trials have excluded patients aged < 12 years and as such, the only experience of complement inhibitor therapy in this group comes from small cohorts or individual cases of compassionate use. Based on these reports, pegcetacoplan has shown signs of similar efficacy to the trial population.^78^^,^^79^ Adding to this body of real-world experience will be informative in the immediate future.

## How Long to Treat Patients With Complement Inhibition

C3G and primary IC-MPGN are chronic diseases. With current treatment, complete remission rates of 14% to 32%^22^^,^^40^^,^^80^^,^^81^ and partial remission rates of 20% to 27%^22^^,^^40^^,^^80^ have been reported in retrospective studies. A higher rate of 66% complete remission was reported in 1 study of children with C3G^24^; however, this rate may have been inflated by inadvertent inclusion of patients with acute postinfectious glomerulonephritis.^82^ Remissions with immunosuppression appear to be more likely in patients with NeF-mediated C3G compared with patients with genetic causes.^83^ It must be noted that current measures of remission rely on proteinuria and eGFR. Although these parameters are established as good proxies for disease activity and outcome, it is known that these diseases tend to follow a remitting-relapsing course where disease flares may occur at any time, which complicates the evaluation of remission.^74^ Relapses are common; in a retrospective study of 97 patients with C3G, 33% who had achieved a clinical remission with corticosteroids and mycophenolate mofetil went on to relapse after treatment discontinuation.^83^

Uncontrolled complement activity in C3G and primary IC-MPGN may lead to progressive kidney lesions and irreversible damage that can contribute to eventual kidney failure. The risk of chronic kidney damage accumulating undetected over time argues in favor of some level of persistent background therapy in these diseases.

#### Outstanding Questions

Discontinuation of complement inhibition in patients with C3G and primary IC-MPGN with excellent response is a strategy that requires more investigation, ideally in prospective studies such as those conducted for eculizumab in atypical hemolytic uremic syndrome.^84^^,^^85^ It would be beneficial for these studies to include an expansive collection of clinical, laboratory, and histopathology data to learn which biomarkers are most beneficial for monitoring patients after therapy withdrawal.

Finally, considering the likely availability of > 1 new complement inhibitor in the near future, clarifying optimal sequential or even combined use strategies of these drugs will be essential.

## How to Monitor Complement Inhibitor Therapy

Kidney Disease: Improving Global Outcomes guidelines recommend regular monitoring of serum creatinine, proteinuria, and urinalysis to identify disease progression.^25^^,^^26^ Serial biopsies have proven sensitive and informative for tracking early disease progression in transplanted patients,^75^ although clearly repeating kidney biopsies in disease management is not ideal and associated with additional challenges in children; so we need other ways to assess ongoing glomerular C3 activation.

#### Outstanding Questions

Markers of complement activity in serum (such as C3, C4, and sC5b-9) are readily available and these may hold some prognostic value, however, their availability is limited and access remains restricted in routine clinical practice.^40^^,^^86^, ^87^, ^88^

Patients presenting with low C3 or high sC5b-9 should be monitored for these biomarkers during treatment and in case of treatment discontinuation. During complement inhibition treatment both biomarkers are expected to normalize, although this may not associate with a clinical response. In both APPEAR-C3G and VALIANT, sustained responses to treatment were observed in circulating biomarkers from baseline to week 26, with an increase in mean serum C3 and a decrease in sC5b-9.^30^^,^^89^ An important clinical question remains: what should the clinician do when a patient receiving a targeted inhibitor shows a reduction in proteinuria below 1 g/d, yet serum complement levels remain low? Currently, there is insufficient evidence to fully understand the clinical implications of incomplete complement inhibition. However, clinical response should prevail. It remains important to monitor these biomarkers, along with glomerular C3, in cases where the clinical response is incomplete. After discontinuation, these complement biomarkers can help determine whether the original trigger for complement dysregulation is still present or has been resolved. Real-world data on complement inhibition will provide further guidance.

Urinary complement markers may offer a promising noninvasive alternative approach in the future^90^; however, the application of monitoring these markers for informing complement inhibitor treatment decisions (i.e. their ability to become biomarkers) needs to be elucidated.

Aside from proteinuria, eGFR, and histology, there are many other potential biomarkers of disease activity whose utility for monitoring during therapy or after therapy withdrawal requires more research. Microscopic hematuria,^91^ C5b-9 deposition on kidney biopsy as a marker of terminal pathway activation,^92^ the degree of NeF-mediated C3 or C5 convertase stabilization,^14^^,^^93^ and the presence of interstitial fibrosis^88^ may all add to the body of evidence required for clinical decision-making.

## Important Next Steps

With potentially practice-changing new complement inhibitors available in C3G and primary IC-MPGN, there will be a need for clinical practice guidelines to incorporate these therapies into their treatment recommendations to support the nephrology community.

Long-term follow-up in clinical trials will be vital to confirming a sustained benefit of different complement inhibitors. Current trials leave a gap for complement inhibition in younger children (aged < 12 years); given the positive outcomes in adolescents from the VALIANT trial of pegcetacoplan,^94^ an open-label, single-arm trial may be sufficient to facilitate treatment access for younger pediatric patients in clinical practice. This represents an urgent unmet need as the disease onset in children often occurs before 12 years of age.^95^

Beyond clinical trials, real-world evidence will add to our understanding of how different complement inhibitors perform in more representative patient populations outside of the ordered environment of a clinical trial, as well as contributing vital long-term safety data. Large international and country-specific disease registries (e.g., the CompCure C3G/IC-MPGN registry within the European Rare Kidney Disease Registry, and many national registries) will play a critical role in collecting high-quality outcomes evidence, with linked biomarker and histology data ideally providing a more granular picture of any potential disease modification. More sophisticated methods of “fingerprinting” patients^96^, ^97^, ^98^ may be useful for investigating nonresponders.

More broadly, with the availability of new therapy options, it will be important to increase awareness among physicians and patients to promote earlier identification of the disease, potentially allowing earlier intervention. Screening programs for proteinuria would be a significant step forward in achieving this goal.

Finally, we are sorely lacking in our understanding of why systemic complement dysregulation specifically affects glomeruli in C3G and primary IC-MPGN. Hypotheses include the complete dependence of the glomerular basement membrane on a single complement regulator (factor H) for appropriate complement pathway function, as well as the high concentrations of complement proteins induced by filtration in the glomerulus. The mesangial inflammatory response may be an important factor, because this kidney compartment too is exposed to plasma proteins and immune complexes during filtration.^99^ More research into these fundamental aspects of disease manifestation may help to refine therapeutic approaches to complement inhibition in the future.

## Conclusion

In a working group, consensus was achieved on proteinuria, eGFR, and histopathological changes as the best current markers of prognosis and treatment efficacy in C3G and primary IC-MPGN.^38^ By these measures, there is no doubt that new complement inhibitors have elicited remarkable responses so far and have the potential to replace current symptom-oriented first-line treatment options, addressing the unmet needs in these rare diseases.

Although some questions still remain around complement inhibitors, including choice of agent and length of treatment, C3G and primary IC-MPGN patients can certainly look forward to a brighter future with meaningful differences to their lives. Continued research, reporting of real-world experiences, and expert guidelines will help to settle outstanding controversies in the coming years.

## Disclosure

DK has received consultancy income from Gyroscope Therapeutics, Alexion Pharmaceuticals, Novartis, Apellis, Sobi, Silence Therapeutics, Roche and Sarepta; and his spouse works for GSK. GA has received honoraria for lectures, educational events, or advisory boards for AstraZeneca (Alexion), Recordati Rare Disease, Advicenne, Chiesi, Kyowa Kirin, Alnylam, Sobi and Dicerna; and served as site investigator for Apellis. MV has received consultancy fees from Novartis, Travere, Roche, Apellis, Sobi, Alexion, BioCryst, Purespring, Bayer, Santhera and WebMD; has participated as local PI in clinical studies funded by Alexion, ChemoCentryx, Bayer, Novartis, Roche, Chinook, Apellis, and Travere; and served on speakers bureaus for Novartis, Sobi, Roche, Vifor, Travere, Alexion and GSK. FS has received consulting fees from Samsung Bioepis and Sobi for participation in Scientific Advisory Board meetings, with payment made to him, and from Alexion for consulting on pediatric trial programs in potential new indications for C5 inhibitors while participating in the Alexion Global aHUS Registry Steering Committee, with payments made to his institution. FC-F has received fees from Novartis, Sobi, Bayer and AstraZeneca. VF-B has received fees from Sobi, Apellis, Alexion, Samsung and Novartis. FF has received consulting fees paid to his institution from Alexion, Apellis, Novartis, Roche and Sobi, and participation on advisory boards for Alexion, Apellis, Novartis, Roche, and Sobi. CL has received consulting fees and honoraria from Alexion, Apellis, Samsung Bioepis, Sobi, Novartis and Pfizer. MCP has received consulting fees from Alexion, Achillion, Annexon, Apellis, Biocryst, ChemoCentryx, Complement Therapeutics, Gemini, Gyroscope, MIRNA Therapeutics, Ormeros, Q32bio Pharma, and Sobi.
